# Cooperative Localization Improvement Using Distance Information in Vehicular Ad Hoc Networks

**DOI:** 10.3390/s19235231

**Published:** 2019-11-28

**Authors:** Felipe Lobo, Danilo Grael, Horacio Oliveira, Leandro Villas, Abdulaziz Almehmadi, Khalil El-Khatib

**Affiliations:** 1 Computer Science Department, Federal University of Roraima, Boa Vista 69310-000, Brazil; 2 Faculty of Business and Information Technology, Ontario Tech University, Oshawa, ON L1G 0C5, Canada; danilo.carvalhograel@uoit.net (D.G.); khalil.el-khatib@uoit.ca (K.E.-K.); 3 Institute of Computing, Federal University of Amazonas, Manaus 69080-900, Brazil; horacio@icomp.ufam.edu.br; 4 Institute of Computing, University of Campinas, Campinas 13083-852, Brazil; leandro@ic.unicamp.br; 5 Department of Information Technology, University of Tabuk, Tabuk 71491, Saudi Arabia; aalmehmadi@ut.edu.sa

**Keywords:** vehicular ad hoc networks, localization systems, data fusion, distance information

## Abstract

In vehicular ad hoc networks (VANets), a precise localization system is a crucial factor for several critical safety applications. The global positioning system (GPS) is commonly used to determine the vehicles’ position estimation. However, it has unwanted errors yet that can be worse in some areas, such as urban street canyons and indoor parking lots, making it inaccurate for most critical safety applications. In this work, we present a new position estimation method called cooperative vehicle localization improvement using distance information (CoVaLID), which improves GPS positions of nearby vehicles and minimize their errors through an extended Kalman filter to execute Data Fusion using GPS and distance information. Our solution also uses distance information to assess the position accuracy related to three different aspects: the number of vehicles, vehicle trajectory, and distance information error. For that purpose, we use a weighted average method to put more confidence in distance information given by neighbors closer to the target. We implement and evaluate the performance of CoVaLID using real-world data, as well as discuss the impact of different distance sensors in our proposed solution. Our results clearly show that CoVaLID is capable of reducing the GPS error by 63%, and 53% when compared to the state-of-the-art VANet location improve (VLOCI) algorithm.

## 1. Introduction

Vehicular ad hoc networks (VANets) require precise localization information, mainly in critical safety-based applications, such as driverless vehicles and blind crossing [1]. However, precise location is a drawback yet that needs to be addressed [2]. To deal with this problem, vehicles are commonly equipped with global positioning system (GPS) devices that provide location information [1,3,4]. However, the accuracy of GPS information can be affected by dense urban areas, such as urban street canyons and indoor parking lots, because of the absence of direct satellite visibility, which turns the GPS into an inaccurate instrument to provide precise location information [5].

To tackle this drawback, there are some solutions proposed in the literature that use anchor nodes [5]. In these approaches, anchor nodes are aware of their positions, so the other nodes can measure their distances using the anchor nodes as references to compute their relative positions [6]. On the other hand, some approaches use a cooperative positioning (CP) technique. These approaches benefit from using vehicle-to-vehicle communication (V2V), in which nearby nodes exchange information about their positions and the relative distance between them and their neighbors [7,8].

Another known technique used to decrease localization error is data fusion [9], which combines location information from different sources to generate a more precise result. In these solutions, data from GPS, geographic information systems (GIS), sensor information, and other sources can be combined using techniques such as particle filter (PF), Kalman filter (KF), or even in linear transformation to estimate more precisely the vehicle’s location [10,11,12]. Nowadays, vehicles come with vehicular safety systems which are composed of several associated sensors, such as cameras, radars, and lasers, to mention a few. So, data fusion techniques can fuse all of this additional information to minimize the GPS error.

In this work, we propose a novel location data fusion technique, called cooperative vehicle localization improvement using distance information (CoVaLID), that cooperatively gathers GPS and distance information from nearby vehicles to improve their locations. Our CoVaLID solution is an extension and improvement over our previously proposed BOuND algorithm [13]. In this current work, we are using a weighted average model in GPS positions to put more confidence in distance information provided by vehicles closer to the target. Thus, we take advantage of these extra sensors to propose a distance-based data fusion technique to improve the localization provided by GPS. Also, we have applied a set of equations based on the concept of congruent triangles. These equations work with information about the difference between both the sensor and the GPS distance. To perform data fusion, we use an extended Kalman filter (EKF) that is fed by results from these equations. Also, we used road constraints to adjust the positions of the vehicles on the road by using only a single anchor node. Thus, we can estimate the new vehicle position through the proposed EKF model.

It is important to note that our solution focuses on GPS inaccuracies, and not on GPS outages since we still need the (possibly inaccurate) position of the vehicles to apply CoVaLID.

Among the contributions of this work we can name:

- our algorithm reaches a high level of accuracy of estimated positions using just GPS data, distance information, and only a single anchor node;
- simulations using different real-world scenarios data such as highway, downtown, and neighborhood;
- an exploratory analysis of the sensors used to provide distance information;
- high level of accuracy of estimated positions even when increased the number of vehicles.

This work is outlined as follows. In Section 2, we present the related work. In Section 3, we describe our proposed solution. In Section 4, we show the methodology, performance evaluation, and obtained results. Finally, Section 5 provides our conclusions and future work.

## 2. Related Work

Some solutions to reduce localization errors and overcome the GPS limitations have been proposed in the literature [14]. In this section, we divide these localization approaches into GPS free and GPS assisted solutions [8].

### 2.1. GPS Free Solutions

In Akcan and Evrendilek [15], the authors propose a novel directional localization algorithm (DWRL) that can compute accurate node localization using distances among nodes without knowing their positions. In this solution, an additional radio is deployed for each node to enable the proposed DWRL algorithm to perform directional localization. This solution has an additional cost due to the need for several additional radios. Another approach that utilizes radios is presented in [16]. The authors proposed localization and synchronization schemes using full-duplex radios. Also, the solution can estimate inter-node distance and clock offset by two consecutive transmissions. However, this solution needs at least three anchor nodes, while our proposed method can be performed using only one anchor node. The solution presented in [17] uses antennas array as well. The authors showed that NLOS components in 5G mm-wave MIMO systems could be exploited to raise position and orientation accuracy. Although, at least three NLOS paths are needed to support the received signal.

On the other hand, in [18] the authors show a proof of concept study that uses vehicle-to-vehicle (V2V) and vehicle-to-infrastructure (V2I) communication as well as an EKF to perform the fusion of time of arrival (TOA) measurements, inertial measurement unit (IMU), and map information to localize a vehicle in GPS-denied environments. Their proposed solution achieved accuracy between 1 m to 5 m with an 11-dimensional vector to describe the vehicle state, which increases the computational cost.

In [19] the authors presented a theoretical foundation of network localization and navigation (NLN) to localize vehicles through anchor nodes. Also, they show a system model composed of inter and intra-node measurements, models for states of Bayesian and non-Bayesian perspectives. To determine the limits of localization accuracy, the authors used an equivalent Fisher information analysis. Furthermore, they identified the influence of antenna arrays and spatiotemporal cooperation on localization efficiency. In our work, we are considering just a Bayesian statistical model. In [20], it was described how operation strategies for NLN, such as node prioritization, node activation, and node deployment can affect localization accuracy. Results show the performance improvement for each one of these strategies. However, the latency of position information was not addressed in their solution.

A geometry-based localization (GeoLV) for GPS outage scenarios was proposed by Kaiwartya et al. [21]. They solved the problem of GPS outage in both short and long outage periods through the equation of a circle and the intersection between the circle and a line to estimate the vehicle’s location more accurately. The GeoLV was tested in three different scenarios, straight, curved, and angular road trajectories. In our proposed solution CoVaLID, we tested the same scenarios presented in [21]. However, unlike GeoLV we used the similarity of triangles concept instead.

A different approach is proposed in [8], in which the authors describe an integrated dead reckoning and cooperative positioning (CP) approach that is capable of locating a vehicle when GPS is unavailable. In their solution, a multihop V2V communication is used to reinitialize Dead Reckoning periodically, when GPS loses their line of sight (LOS) with satellites. Moreover, the geometric dilution of precision (GDOP) concept is applied to obtain the best combination of nodes to operate the multilateration technique. However, the authors did not try any prediction models Bayesian statistics-based, such as Kalman filter, to improve the accuracy of their solution.

### 2.2. GPS Assisted Solutions

Apart from the solutions presented previously, there are also solutions assisted by GPS, in which the GPS information is combined with other techniques and information such as the received signal strength Indicator (RSSI) and the time of arrival (ToA). Among these solutions, we can highlight a few. For instance, Suryawanshi et al. [22] proposed a system based on a path detection algorithm (PPD), which improves the inter-vehicular communication assisted location (IVCAL). This approach uses GPS as the primary measurement for the position of the nodes while using Kalman Filter to reduce the signal noise output, to identify the previous path. In our proposed solution, the CoVaLID algorithm uses V2V communication between the nodes to exchange information about both vehicle position and distance among nearby vehicles. However, our focus is to improve the location information instead of identifying the path.

Golestan [23] demonstrated two Data Fusion methods using GPS data, wheel sensors information, gas and brake pedal, and V2V communication. In the first method, the authors measured the belief of each vehicle related to its current location using the extended Kalman filter (EKF). In their second method, they extended a particle filter (PF) to weight the particles equivalent to the beliefs. Their results show a high accuracy of 1.65 m of mean absolute error (MAE) values. However, the methods can only reach that accuracy when the number of vehicles is at least five.

Another interesting GPS assisted method is presented by Farhan [24], where the authors proposed the VLOCI algorithm. Similar to our solution, vehicles exchange GPS position information. Also, they assume that all vehicles are capable of measuring the distances among themselves. They also consider that vehicles are traveling in one lane and following the same direction. Thus, the distance information is used to improve the position only in one axis. On the other axis, they assume there is no error since vehicles are moving in a straight-line trajectory. After the GPS data exchange, the VLOCI algorithm is executed, and a set of neighbors coordinates is computed. A weighted average technique is applied to use the more reliable information from closer vehicles while giving less priority to further vehicles. As a result, the best MAE value was of 2.38 m, and at least 5 vehicles are needed to reach this accuracy. It is worth mentioning that, despite its limitations, the VLOCI is a state-of-the-art localization technique that uses only GPS and V2V communication [25]. For this reason, we chose the VLOCI algorithm to compare with our proposed solution. Algorithm 1 summarizes more clearly how the VLOCI algorithm works.

**Algorithm 1** VLOCI algorithm. **while** iteration < I **do**  transmitMessage($\hat{p}i$) *M* contain received messages  **for** each item $M_{j}$ in *M*
**do**   ${\hat{d}}_{i,j}=\frac{1}{D}\sum_{k=1}^{D}takeDistMeas\left(n_{j}\right)$   **if**
${\hat{x}}_{i}$ < ${\hat{x}}_{j}$
**then**    $p_{i}^{j}=\left({\hat{x}}_{i}^{j},{\hat{y}}_{i}\right)=\left({\hat{x}}_{j}-{\hat{d}}_{i,j},0\right)$   **else**    $p_{i}^{j}=\left({\hat{x}}_{i}^{j},{\hat{y}}_{i}\right)=\left({\hat{x}}_{j}+{\hat{d}}_{i,j},0\right)$  ${\hat{x^{\prime }}}_{i}=\frac{\sum w\left(n_{k}\right)\cdot {\hat{x}}_{i}^{k}}{\sum w(n_{k})}\cdot {\hat{y^{\prime }}}_{i}=0$

Our proposed solution is also based on the cooperative exchange of GPS data. The CoVaLID algorithm is a localization technique capable of adjusting the vehicle GPS coordinates based on inter-vehicular distance data. It relies on accurate distance information that is given by measurement devices such as lidar, radar, or cameras. To perform the data fusion of GPS and distance information, we applied an extended Kalman filter (EKF), which is different from the VLOCI. Another noticed difference is that CoVaLID is designed to improve GPS coordinates in both axes, whereas VLOCI can improve just in one axis. Therefore, the main contribution of this work is that we can reach a high level of accuracy of the estimated positions using only GPS and distance information, which has a low computational cost due to the Kalman filter recursive call.

## 3. CoVaLID Localization

In this section, we describe the details of our proposed solution, the CoVaLID (Cooperative Vehicle Localization Improvement using Distance Information) algorithm which is an extension and improvement over our previously proposed BOuND algorithm [13]. We also explore and discuss some of its challenges and real-world implementation.

### 3.1. Problem Statement

In this work, to simplify the localization problem, we take into account only two dimensions (2D). However, our proposed solution is also suitable for three dimensions (3D) and could be easily adapted.

**Definition** **1.** *Let $X=[X_{1},\ldots ,X_{N}]$ be a set of position coordinates, $\forall X_{i}\in R^{2}$, in a two dimensional plane, where N is the number of vehicles, and $\langle X_{i},X_{j}\rangle \in X$ if $X_{i}$ is in the communication range of $X_{j};\;\forall X_{i}\in X,\;X_{i}=\left[x_{i},y_{i}\right]$ is the position coordinates of vehicle i, given by the GPS;*

**Definition** **2.** *The GPS accuracy can be affected by some factors, such as atmospheric conditions, satellite positions, and natural barriers to the signal, to cite a few [26]. Given a set of vehicles V, where each vehicle $v_{i}$ has its GPS position $Gp_{i}$, and its true position is $Tp_{i}$, the GPS error ($E_{gps}$) is defined as:*

(1) $$E_{gps}=||Gp_{i}-Tp_{i}\left|\right|$$

It is important to mention that the bigger $E_{gps}$, the bigger the GPS distance information error. Thus, our CoVaLID solution is directly affected since GPS distance information is used in Equation (5) results. These results are a key feature in our proposed solution since it is used to compute the new estimated vehicle position through the concept of similarity of the triangles. However, in our solution, this problem is minimized, as shown in Section 4.3.2.

**Definition** **3.** *The Euclidean Distance between two vehicles $\left(V_{i}\right)$ and $\left(V_{j}\right)$ is given by:*

(2) $$d_{i,j}=\sqrt{{\left(x_{i}-x_{j}\right)}^{2}+{\left(y_{i}-y_{j}\right)}^{2}}$$

*where $\left(x_{i},y_{i}\right)$ and $\left(x_{j},y_{j}\right)$ are respectively, the inaccurate position of vehicles $V_{i}$ and $V_{j}$, given by the GPS, and $d_{i,j}$ is the distance between them.*

**Figure 1:**
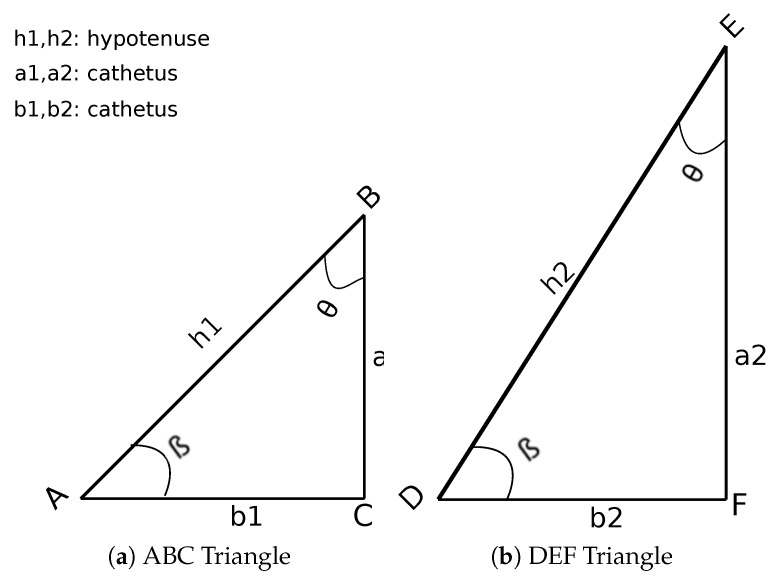
Two triangles with different sizes but sharing congruent angles (β and θ) are similar.

**Definition** **4.** *The concept of similarity of the triangles as demonstrated in our previous work [13], states that if two triangles share congruent angles, they are similar, as shown in Figure 1. Hence, the ratios of the corresponding sides of any two triangles are equivalent, no matter the hypotenuse length.*

(3) $$\frac{h_{1}}{h_{2}}=\frac{a_{1}}{a_{2}}=\frac{b_{1}}{b_{2}}=c,$$

*where c is the constant of proportionality, h, a and b are the sides of the triangles.*

Due to the demonstrated property above (similarity of the triangles), it supports that the ratio of two sides in one particular triangle is equal to the ratio of two sides in another similar triangle. From Equation (3) we can formulate:

(4) $$\frac{a_{1}}{h_{1}}=\frac{a_{2}}{h_{2}}.$$

**Definition** **5.** *Since the GPS can provide noisy coordinates, we can compute the difference of the distances between both that information given by the sensor and the one given by the GPS. Hence, we denoted distance error as:*

(5) $$tD=Dist_{Gps}-Dist_{Sensor}.$$

In this work, we are taking into consideration that near vehicles have related GPS errors. Although the different brands of GPS receptors do result in different errors, it is known that they are spatially auto-correlated, which means that vehicles in similar locations have similar errors [27]. However, it is worth to mention that real-world errors were introduced in our simulation environment to model the difference in GPS receivers brands.

### 3.2. Applying the Concept of Similarity of the Triangles

The CoVaLID localization technique is constituted of two equations, the same used in BOuND [13]. We can obtain these equations as follows:

Equation (2) gives us the distance information using the GPS coordinates. Whereas, Equation (5) provides the difference between both the GPS distance and the sensor distance.

**Figure 2:**
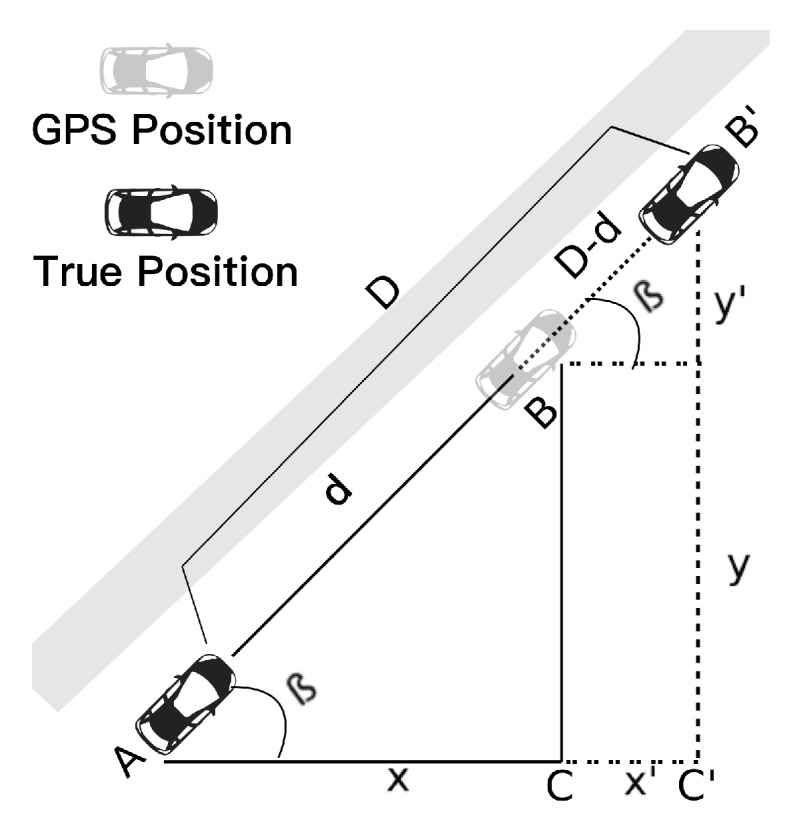
Vehicles communicating with each other via vehicle-to-vehicle (V2V)—exchange information about its own GPS location and vehicle *A* sending the distance information given by sensors.

Figure 2 shows that *D* is the sensor distance information. As explained in the next section, we are using the weighted average information. Here, *d* is the distance computed based on the GPS positions of both vehicles A and B. Once we have this information, we can calculate the difference (D−d) of the distance between the vehicles, the coordinates of vehicle B, centering in-vehicle A, are given as *x* and *y*. Moreover, using the concept of similarity of triangles, we can notice that the β angle is the same in both triangles ACB and triangle $AC^{\prime }B^{\prime }$. Hence, CoVaLID can adjust the vehicle’s position based on the difference between the sensor and the GPS distances. With all the needed information, we can utilize the concept of similarity of the triangles to estimate the new vehicle position, through Equations (6) and (7).

(6) $$\frac{D-d}{x^{\prime }}=\frac{d}{x}$$

(7) $$\frac{D-d}{y^{\prime }}=\frac{d}{y},$$

where the $x^{\prime }$ and $y^{\prime }$ are the residual values that must be used to estimate the new vehicle coordinates. The Equations (6) and (7) can be derived in:

(8) $$x^{\prime }=\frac{(D-d)x}{d}$$

(9) $$y^{\prime }=\frac{(D-d)y}{d},$$

finally, we can obtain the new estimated coordinates through:

(10) $$X_{new}=X_{gps}+x^{\prime }$$

(11) $$Y_{new}=Y_{gps}+y^{\prime }.$$

It is worth mentioning that we assume the error in both the *x*-axis and the *y*-axis is proportional, which might not be accurate in some real-world scenarios. Moreover, CoVaLID can be used in real-world scenarios, despite its use of straight lines. For instance, if two vehicles are on the same road (straight line), the sensors can collect distance information even if they are not in the same lane which is a fair assumption since both highways and downtown scenarios are common scenarios.

**Figure 3:**
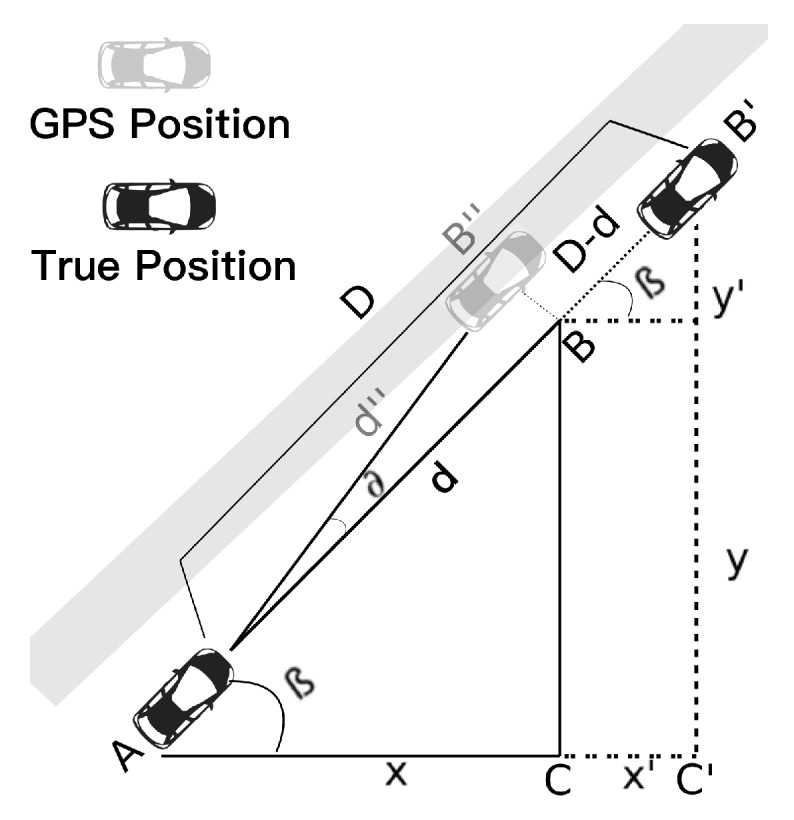
Vehicles communicating with each other via V2V—exchange information about its own GPS location and vehicle *A* sending the distance information given by sensors.

Sometimes the GPS position may not be on the same line as the one formed by the true positions of vehicles *A* and $B^{\prime }$, as shown in Figure 3. In these cases, we can compute the GPS distance ($d^{\prime \prime }$) between *A* and $B^{\prime \prime }$. Also, we still have distance information from both the GPS and the sensor (*D*), so we can use CoVaLID. Thus, we assume the GPS position is in the same line as the one formed by the true positions of *A* and $B^{\prime }$. Thus, the distance value $d^{\prime \prime }$ is equal to *d*, which may not be true in the real world. However, the less the angle *∂*, the closer $d^{\prime \prime }$ will be regarding *d*. Hence, we performed our algorithm as the GPS position was in the same line of the sensor’s position, as seen in Figure 2.

### 3.3. Gathering Distance Information

As aforementioned, each vehicle sends its GPS position along with the distance information every second. Thus, the target vehicle, when it receives the neighbors’ information can perform a weighted average on its GPS position and use the sensor’s distance information along with the similarity of the triangles method for each pair of vehicles. It is worth mentioning that in this work, we are focused only on distance information that is given by sensors, such as cameras, lasers, or radars. Hence, how these sensors gather this information is not our focus.

Also, we can notice in our previous work [13] that the vehicles farther away from the target can provide less accurate distance information than closer vehicles. The main idea in this work is to put more weight in the distance information given by neighbors closer to the target and less weight for the ones that are farther. In the VLOCI algorithm [24] the authors compute the weighted average for the target’s GPS position received from its neighbors using Equation (12).

(12) $$x^{\prime }=\frac{\sum_{i=1}^{n}w_{i}x_{i}}{\sum_{i=1}^{n}w_{i}},$$

where $x_{i}$ are the GPS coordinates, and $w_{i}$ is its respective weight.

We compute the weighted average for the target vehicle’s position in the same way as in [24]. Hence, this weighted average position is used to provide the GPS distance information. However, differently from VLOCI, we use different weights for each distance range, according to Table 1. As a result, we get this distance information to feed our extended Kalman filter. It is worth mentioning that if there is more than one node in the same range, it distributes equally the weights for them. Also, the values showed in Table 1 are based on the results of our earlier work [13].

### 3.4. Extended Kalman Filter

A Kalman Filter or one of its derivatives can be a suitable method to perform data fusion. The KF is used as a filtering component based on an iteration process that is divided into two phases: a prediction and an update phase [28]. Moreover, it is an optimal linear estimator for Gaussian noise. Also, it can be used even with non-linear systems due to its variations such as the extended Kalman filter (EKF) that can linearize the problem by calculating its partial derivative. Due to our proposed solution nature, we implemented an EKF, and it is fed by both the GPS coordinates, corrected by Equations (10) and (11), and the sensor distance information.

The EKF prediction phase uses the information from the last time step to produce an estimated state at the current time step, as seen in Equations (13) and (14):

(13) $$x_{k}=F_{k}x_{k-1}+B_{k}u_{k}$$

The predicted error covariance is calculated by:

(14) $$P_{k}=jFP_{k-1}jF^{T}+Q_{k},$$

where $F_{k}$, is the transition matrix; the state matrix x=[xA,yA,xB,yB] that are the estimated coordinates of the pair of vehicles *A* and *B*, given by the similarity of triangles method; $x_{k-1}$, is the observation matrix; the covariance of the process noise is $Q_{k}$; the $B_{k}$ is the input control matrix model applied over vector *u*; $P_{k-1}$ is the initial uncertainty in the process. Finally, both jF and $jF^{T}$ are the Jacobian matrix of the state matrix and its transpose, respectively.

In the second phase, the update is given by the set of equations as follows. The measurement matrix *z* is composed of GPS positions of vehicles *A* and *B*, and the true distance information (the gathering of distance information):

(15) z=[Ax,Ay,Bx,By,trueD].

The sensor readings are expressed as the measurement matrix $H_{k}$. However, the relationship between the measurements and the state vector is required. To meet that requirement, we can observe two interesting points. First, the GPS measurements have a linear relationship with the state vector, since GPS provides the coordinates of both axes. Second, the distance of the sensor measurements is gathered in polar coordinates, which means that we need to convert them from polar to Cartesian coordinates in the matrix below.

(16) $$h\left(x^{\prime }\right)=\left\{\begin{array}{c} x\\ y\\ \sqrt{x^{2}+y^{2}} \end{array}\right\}$$

It is noticed that the problem described in the matrix above is non-linear, so we can apply the EKF to linearize it. For that purpose, the Jacobian (partial derivative) is used to estimate $jH_{k}$ (the Jacobian matrix of $H_{k}$), and $jH_{k}^{T}$ is its transpose. The measurement’s noise is given by *v*, which is assumed to be zero-mean Gaussian white noise with covariance $R_{k}$. Hence, the Kalman gain can be calculated by:

(17) $$K=P_{k|k-1}jH_{k}^{T}{\left(jH_{k}P_{k|k-1}jH_{k}^{T}+R_{k}\right)}^{-1}.$$

Furthermore, the difference between the measurement and state estimation *y* can be obtained by:

(18) $$y=Z^{T}-\left(jH_{k}\cdot x_{k}\right).$$

Consequently, the formulas for update both the uncertainty process covariance and the estimation state are expressed by:

(19) $$P_{k}=\left(I-K_{k}jH_{k}\right)P_{k|k-1},$$

where *I* is the identity matrix.

(20) $$x_{k}=x_{k|k-1}+\left(K\cdot y\right).$$

Thus, the coordinates are estimated by the EKF and they sometimes can result in an off-road position. So, they need to be adjusted according to the road boundaries.

### 3.5. Adjusting Vehicle Position

To adjust the vehicle position, we compare the new estimated vehicle position computed by EKF with the path geometry of the road. We use map information to restrict the estimated vehicle position onto the identified road. Moreover, we assumed that our proposed solution has access to a digital road map. Thus, we can verify if the vehicle’s estimated position is within the road limits. If that is not the case, the algorithm shifts the vehicle position to the nearest point onto the road.

## 4. Simulation Results

### 4.1. Methodology

To evaluate the behavior of our proposed solution, we have used the simulation of urban mobility (SUMO) [29] for scenario construction, Omnet++ [30] along with Veins framework [31] for vehicles communication and python scripts for statistical computing. Hence, it was possible to define all vehicle mobility and all vehicular network parameters according to the IEEE 802.11p standard. The parameters used in the simulations are described in Table 2.

Concerning the network topology, we took into account that all vehicles are inside their communication range. Thereby, each vehicle is capable of communicating with each other. Thus, vehicles can exchange both their location information given by GPS and the sensor distance information. When one vehicle receives this information, it can start the computation process by constructing the needed matrices and computing the proposed method CoVaLID.

Moreover, to compare the approaches fairly, we had to make some adjustments to the VLOCI algorithm since in the original approach, the network is static, i.e., vehicles were set to be stationary. Thus, in our simulations, all vehicles were set up with constant velocity in an intersection scenario, and the number of iterations was the same as used in [24]. Hence, we considered that all the vehicles had an acceleration equal to zero. Also, the target vehicle is the one in the front, and its neighbors are lined up, and lastly, their trajectories were defined in the north/south direction.

We evaluate the accuracy of our proposed solution related to the impact of three different aspects. First, concerning the number of vehicles, to verify the behavior of the presented solution, we used multiple increasing values. Second, to evaluate the impact of the trajectory on the accuracy over the tested approaches, it was divided into two parts, straight-line and curve. Finally, to verify how the noise in distance measurements can affect the proposed solution, we evaluated the impact of distance information error.

### 4.2. Analysis of the Error

To evaluate our proposed solution, we conducted an analysis using the root-mean-square-error (RMSE) method, described in (Equation (21)). This metric is commonly used to measure the error of the localization approaches.

(21) $$RMSE=\frac{1}{n}\sum_{i=1}^{N}{\left(x-x^{\prime }\right)}^{2}+{\left(y-y^{\prime }\right)}^{2},$$

where (x,y) and $\left(x^{\prime },y^{\prime }\right)$ are respectively the perfect and estimated vehicles’ positions, while the latter varies between GPS, VLOCI, and CoVaLID.

Furthermore, we used the mean absolute error (MAE) as a metric to evaluate our method since some works in literature [24,32,33,34] also use it to assess their results.

(22) $$MAE=\frac{1}{n}\sum_{i=1}^{N}\left|\left(x-x^{\prime }\right)\right|,$$

where (x) and $\left(x^{\prime }\right)$ are respectively the perfect and estimated vehicles’ coordinates, while the latter varies between GPS, VLOCI, and CoVaLID. In Equation (22), we compute the MAE for one axis to simplify the explanation. However, it is suitable for as many axes as necessary.

### 4.3. Simulation Scenario

In this section, we used a simple intersection scenario to evaluate the performance of our proposed localization solution. In this scenario, vehicles can move in a straight-line road. Furthermore, we used RMSE and MAE to assess the accuracy of the VLOCI, CoVaLID, and GPS regarding the impact of GPS error, the increasing of the number of vehicles, and distance among vehicles. Then, both the results and discussion about them are presented.

#### 4.3.1. Accuracy Evaluation

In this section, we compare the results of our proposed solution to the initial GPS inaccurate coordinates, to the VLOCI algorithm, and also to the perfect position of vehicles. For this, we plotted graphs with vehicles’ positions as a result of each cited approach. In these graphs, the yellow circle represents GPS position, whereas the cyan cross, the red cross, and the blue line denote, respectively, CoVaLID, VLOCI, and the ground truth position.

**Figure 4:**
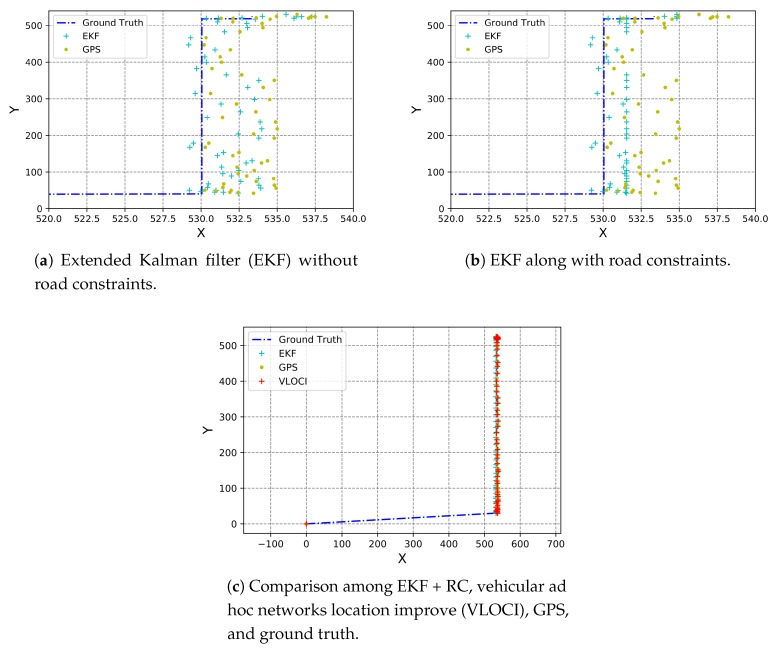
Comparison among CoVaLID, CoVaLID+RC VLOCI, GPS, and ground truth.

As shown in Figure 4a, our proposed solution was able to improve the GPS positions. However, sometimes, those estimations still put the vehicle outside the road. So, we apply the road constraints, as described in Section 3.5, in our data fusion solution, resulting in a more accurate estimation, as seen in Figure 4b. Also, it is noticeable that the trajectory of the vehicle using CoVaLID + RC is similar to the ground truth. According to Table 3, the CoVaLID + RC, called just CoVaLID from now on, is capable of reducing *x*-axis and *y*-axis GPS positioning error on average in 62% and 22%, respectively. Another interesting point in Table 3 is that the VLOCI algorithm had better performance when compared to CoVaLID without road constraints (RC). It can be explained due to the fact that we made some adjustments in the VLOCI original approach, and one of them was to use road constraints. So, VLOCI was already using RC, while CoVaLID not.

We noticed, for this scenario, that the VLOCI algorithm improved its accuracy when compared to the results presented by Farhan [24] due to the adjusts that we made. It is important to mention that the VLOCI approach assumes that vehicles are traveling in one lane and in the same direction. Hence, the values in both, RMSE and MAE are the same in the *x*-axis for VLOCI and GPS techniques. So, when comparing our CoVaLID solution to the VLOCI, in terms of accuracy in the *y*-axis, our approach outperforms VLOCI by at least 11%, reducing the error from 1.85 m to 1.64 m. We can also observe differences between values when the axis changes. It can be explained due to the fact that we assume the error in both axes is proportional, which may not true in real-world scenarios.

#### 4.3.2. The Impact of GPS Error

To study the impact of GPS error regarding the accuracy of the tested solutions, we varied the GPS error parameter by 1, 5, and 10 m, respectively.

**Figure 5:**
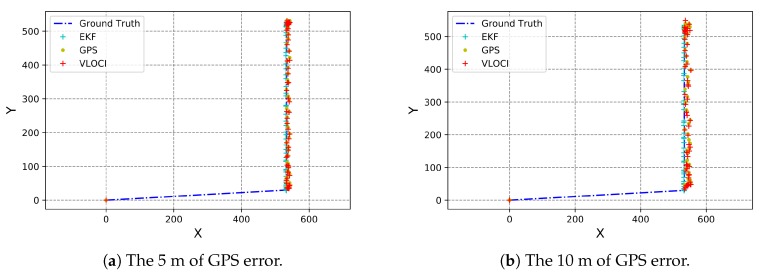
Impact of GPS error—increasing the GPS error from 5 to 10 m.

We can see that in the one-meter GPS error scenario, the CoVaLID, VLOCI, and GPS trajectories are almost the same as the ground truth, as shown in Figure 5a. However, our proposed solution is slightly better when compared to the other techniques. When the GPS error increased to 5 and 10 m, respectively, both CoVaLID and VLOCI could still reduce and improve the GPS localization. Besides, our proposed solution, CoVaLID reached its best performance in 10 m of GPS error scenario, minimizing it on average of both axes in 58% when compared to GPS, and 51% when compared to VLOCI. It is worth mentioning that when the GPS error increases, the trajectory of VLOCI algorithm is quite different than the ground truth, as seen in Figure 5b, while the CoVaLID maintained its trajectory similar to the ground truth.

In Table 4, Table 5 and Table 6, we can notice that our proposed solution obtained the least RMSE values in all cases when compared to both GPS and VLOCI.

We can also notice that albeit VLOCI had improved its performance when the GPS error increased from 1 to 10 m, the algorithm depends on the GPS accuracy, in other words, the more accurate the GPS device is, the more efficient VLOCI can be. Our proposed solution demonstrated similar behavior since it is also a GPS assisted approach. However, CoVaLID shows to be efficient in all evaluated scenarios.

#### 4.3.3. The Impact of Number of Vehicles

To assess the impact of the number of vehicles in all tested approaches, we kept the GPS error constant at 2 m, while the number of vehicles was increased from 2 to 10. Furthermore, we maintained the distance constant among all neighbors regarding the target vehicle in 30 m. To evaluate the performance of each technique, we took into account both the RMSE and MAE values regarding the *x*-axis and *y*-axis, separately, as well as the average between both axes. All graphs presented in this section were plotted with 95% confidence interval.

**Figure 6:**
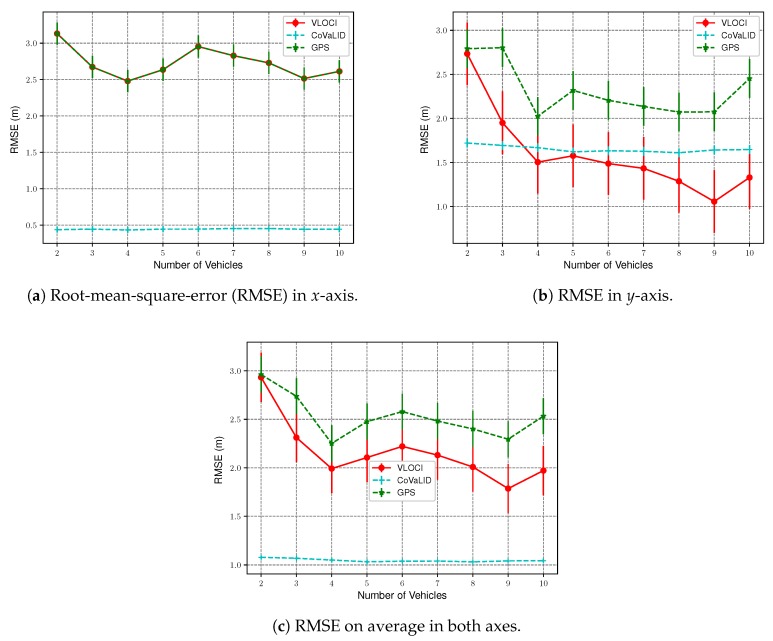
RMSE Values in *x*-axis, *y*-axis, and on average in both axes—regarding the increase in the number of vehicles.

**Figure 7:**
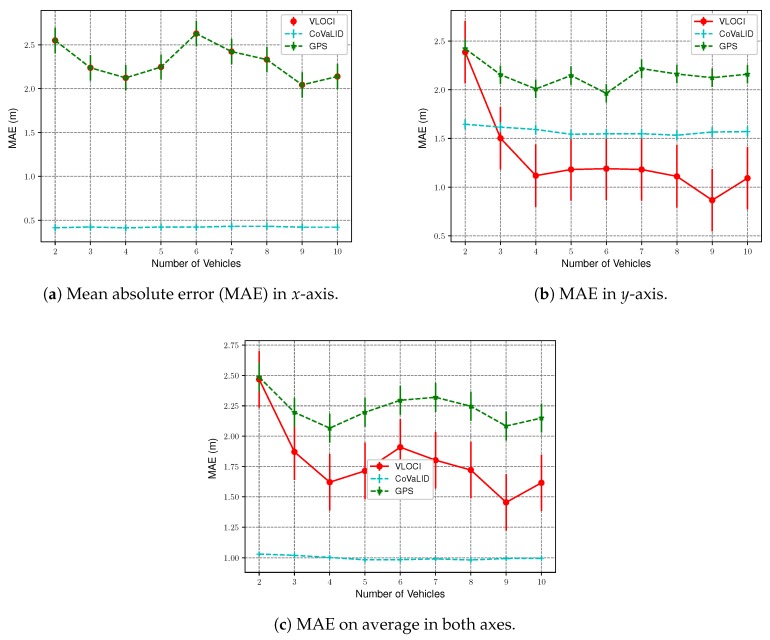
MAE Values in *x*-axis, *y*-axis, on average in both axes—regarding the increase in the number of vehicles.

The RMSE and MAE values show that our proposed method had better performance in all evaluated scenarios when compared to both VLOCI and GPS regarding the *x*-axis. This result is expected since in *x*-axis both have the same values, as presented in Figure 6a and Figure 7a.

Another interesting point is that when the number of vehicles increases to 3, MAE values demonstrated that the VLOCI algorithm could overcome CoVaLID regarding the *y*-axis, as shown in Figure 7b. However, according to RMSE values, the VLOCI algorithm overcomes our proposed method only when the number of vehicles is increased to 4, and maintained its better performance for the remainder of the tested scenarios, as seen in Figure 6b. It suggests that when the number of vehicles increases, better accuracy is achieved in the *y*-axis by VLOCI. Also, it is worth pointing out that although our solution was overcome by VLOCI when the number of vehicles increased, our method maintained RMSE and MAE values almost constant.

Figure 6c and Figure 7c show the average error of both axes. We can notice that our proposed method had better results in all tested scenarios when compared to both VLOCI and GPS. It can be explained by the fact that CoVaLID uses distance information to minimize the GPS error in both axes, while the VLOCI algorithm only improves the error in one axis.

Overall, the results support that our method can be used to circumvent the real-time position estimation problem in VANets using fewer vehicles than the VLOCI algorithm.

#### 4.3.4. The Impact of Distance Values

To evaluate the impact of the distance between two vehicles in the RMSE and MAE values, we kept the GPS error at 2 m and increased the distance between them. The distance values used in this scenario were: 11.8, 23.7, 35.6, 47.5, and 59.4 m. All graphs presented in this section were plotted with a 95% confidence interval.

**Figure 8:**
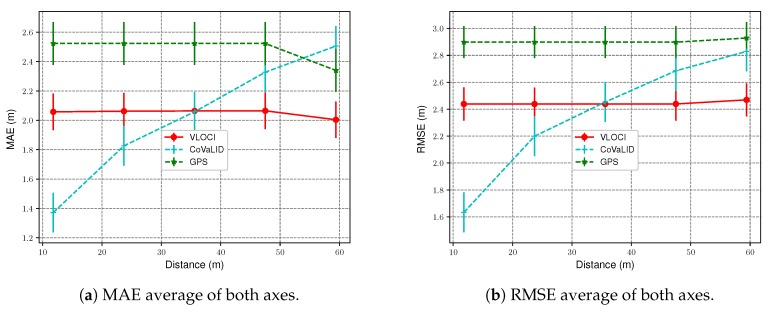
MAE and RMSE values of the average of both axes—regarding the increase of distance values.

Figure 8a,b show the RMSE and MAE values of the average of both axes. We can notice that CoVaLID is directly affected when the distance between neighbors increases. However, our proposed approach had better performance when compared to the VLOCI for vehicles near the target. Although, for long distances between the vehicles, more specifically when the range is greater than 35 m, the VLOCI overcomes our proposed solution.

An interesting point that we can observe is that when increased distance values, the VLOCI algorithm is not affected. This fact can be explained because this algorithm uses the weighted average technique. VLOCI puts more weight in small distance values while putting less weight for higher distances, which means that VLOCI is capable of keeping its performance constant even with different distances between the target vehicle and its neighbors.

### 4.4. Real World Scenarios

**Figure 9:**
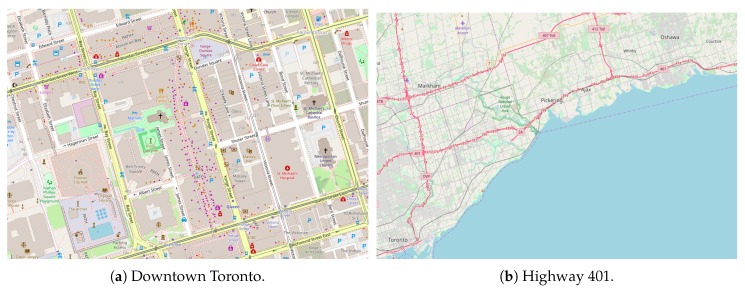

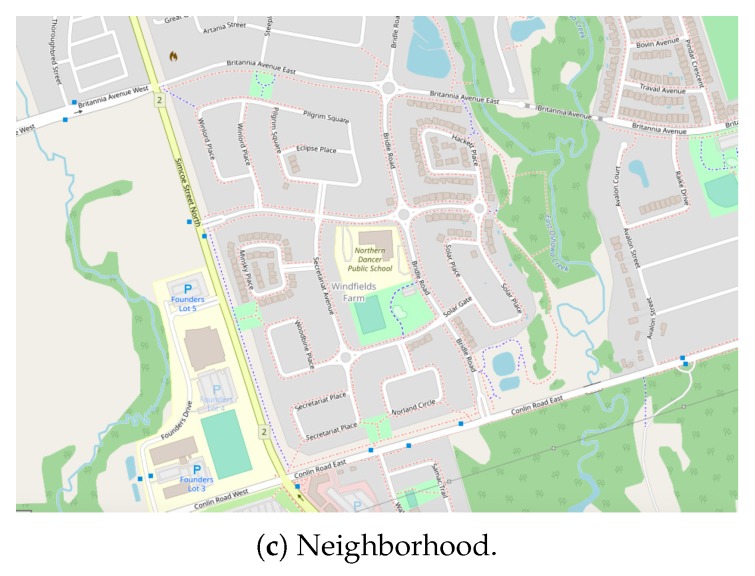
Real world scenarios.

This section aims at evaluating the CoVaLID, VLOCI, and GPS methods in real-world scenarios. Thus, we used three different scenarios: downtown, highway, and neighborhood. The Figure 9a shows Downtown Toronto, which we took as scenario the most famous streets with a considerable amount of traffic, such as Dundas St., Yonge St., Church St., Queen St. and Bay St. In order to use a highway scenario, we chose Highway 401 (seen in Figure 9b) which has heavy traffic once it does not charge tolls. Finally, as the neighborhood scenario, we took into account the one named Windfields Farm that is close to the UOIT north campus, as we can notice in Figure 9c.

Furthermore, since the RMSE and MAE values demonstrated similar behavior, we will only use the RMSE values to assess the accuracy of the VLOCI, CoVaLID, and GPS regarding the impact of increasing the number of vehicles, the distance information error, and the distance among vehicles in simulation scenarios. From now on, we are using our proposed solution CoVaLID along with the weighted average from Section 3.3. Also, as before, all graphs presented were plotted with a 95% confidence interval.

#### 4.4.1. The Impact of Number of Vehicles

In this section, we kept the GPS error constant at 2 m, while the number of vehicles was increased from 2 to 10. Furthermore, both the distance among vehicles and vehicles’ velocities were set randomly. To evaluate the performance of each technique, we took into account both the RMSE values regarding the *x*-axis, the *y*-axis, as well the average between both axes.

**Figure 10:**
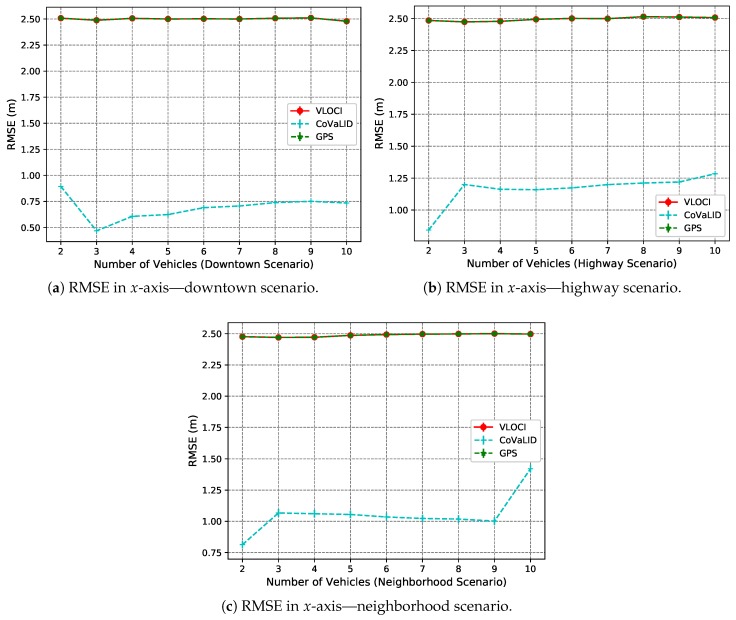
RMSE Values in *x*-axis in downtown, highway, and neighborhood scenarios—regarding the increase in the number of vehicles.

As we can see in Figure 10a–c the VLOCI and GPS had the same value as explained in Section 4.3.1. Furthermore, we can notice that the CoVaLID had its best performance regarding the *x*-axis in the downtown scenario, while in the highway, it performed with accuracy almost constant, as well in neighborhood scenario, except when increased the number of vehicles for 10. The best accuracy in the downtown scenario can be explained because, in a highway scenario, the vehicle velocity is higher. Hence, the higher the velocity, the more affected is our proposed approach in the *x*-axis.

**Figure 11:**
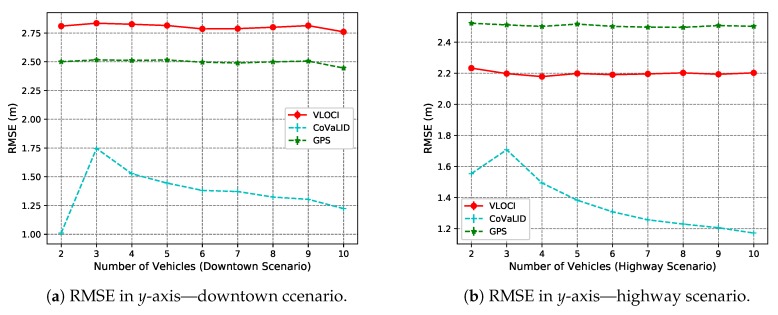

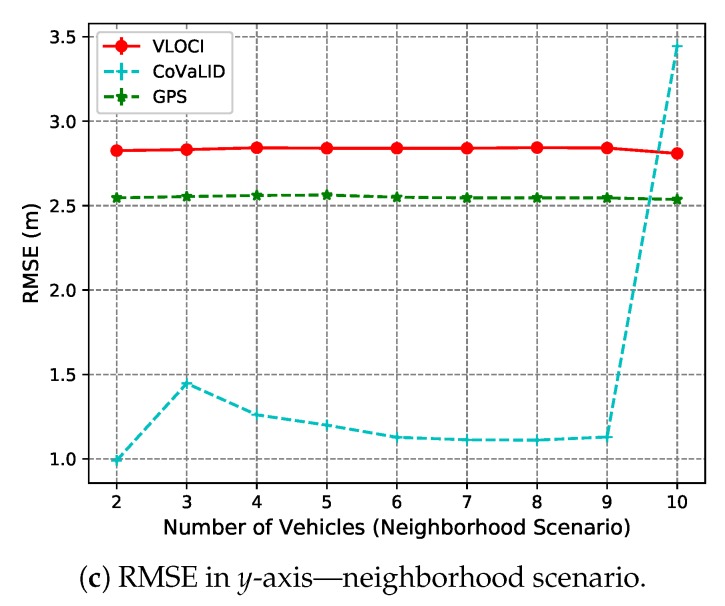
RMSE values in *y*-axis in downtown, highway, and neighborhood scenarios—regarding the increase in the number of vehicles.

In the *y*-axis, according to RMSE values described in Figure 11a–c, our proposed method had better performance when compared to both VLOCI and GPS in downtown, highway, and until 9 vehicles in neighborhood scenario. However, when the number of vehicles increased to 10, it can be noticed that CoVaLID had its performance significantly affected. It is explained because, in scenarios with turns, it is more challenging to apply the similarity of triangles concept, since the communication can be affected by obstacles, such as buildings, and houses. Another interesting point is that contrarily to the *x*-axis, we can notice is that the higher the velocity, the less affected is our proposed approach in the *y*-axis. Also, in both downtown and neighborhood scenarios, we can notice that VLOCI had the worst performance, it can be explained because VLOCI was developed and tested in straight-line scenarios that is one of the characteristics of highway scenario, where VLOCI can overcome GPS accuracy.

We can notice that when the number of vehicles increases to 3, according to RMSE values, the CoVaLID algorithm had a slight decreased in its accuracy, which can be explained due to the use of random distance among vehicles, as well some obstacles and turns during the trajectory. These factors can affect our proposed solution since it assumes that the distance information is perfect. In other words, it does not take into account noise in distance information, which is not true in real-world scenarios. Furthermore, the accuracy of the distance information depends on which sensor is used.

**Figure 12:**
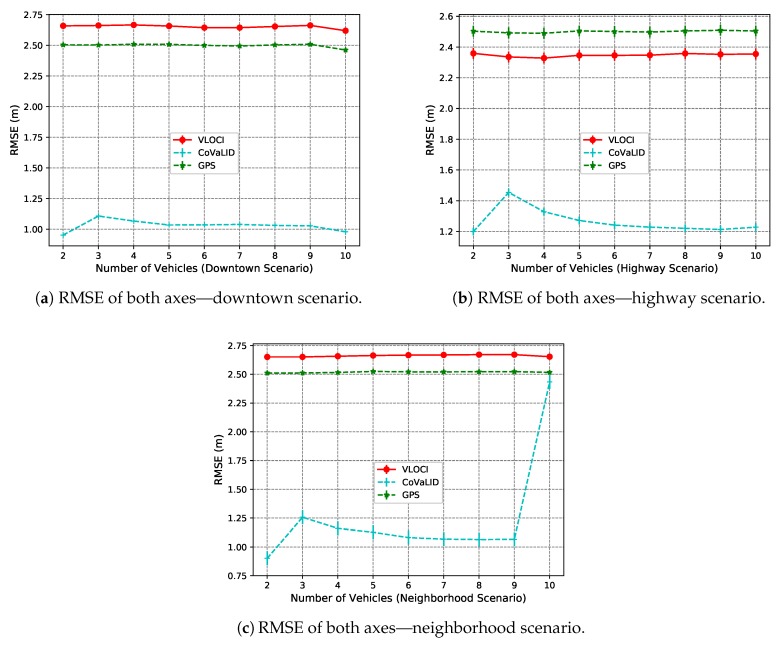
RMSE values of the average of both axes in downtown, highway, and neighborhood scenarios—regarding the increase in the number of vehicles.

Another interesting point is that when increasing the number of vehicles, the CoVaLID performance improves due to the use of the weighted average method of nearby vehicles’ positions. Also, the results suggest that CoVaLID can be used as a solution for localization problem aided by GPS in all tested scenarios, except when the number of vehicles is increased to 10 in neighborhood scenario. In this particular case, the RMSE values, as seen in Figure 10c, Figure 11c and Figure 12c, show that CoVaLID had the worst performance due to the 10th vehicle being farther to the target and as a consequence, its distance information become noisy, since in neighborhood scenarios there are only one-lane streets and sometimes the 10th vehicle is not even in the same street as the target vehicle. However, on average of both axes, as seen in Figure 12a–c, results suggest that our proposed solution is suitable for all tested scenarios. However, in the neighborhood scenario, CoVaLID presented limitations on its performance, when used with 10 vehicles.

#### 4.4.2. The Impact of the Vehicle Trajectory

In this section, we divided the target vehicle trajectory into two parts: when vehicles are in a straight line or when they are in a turning scenario. In addition, we kept the GPS error constant at 2 m. We also used two vehicles, and the distance between them was set at 5 m apart. Thus, we can evaluate the impact of the vehicle trajectory regarding the accuracy of tested approaches in real-world scenarios.

Furthermore, each one of the three real-world scenarios was divided into a straight-line and turning scenarios, as described in Appendix A.

**Figure 13:**
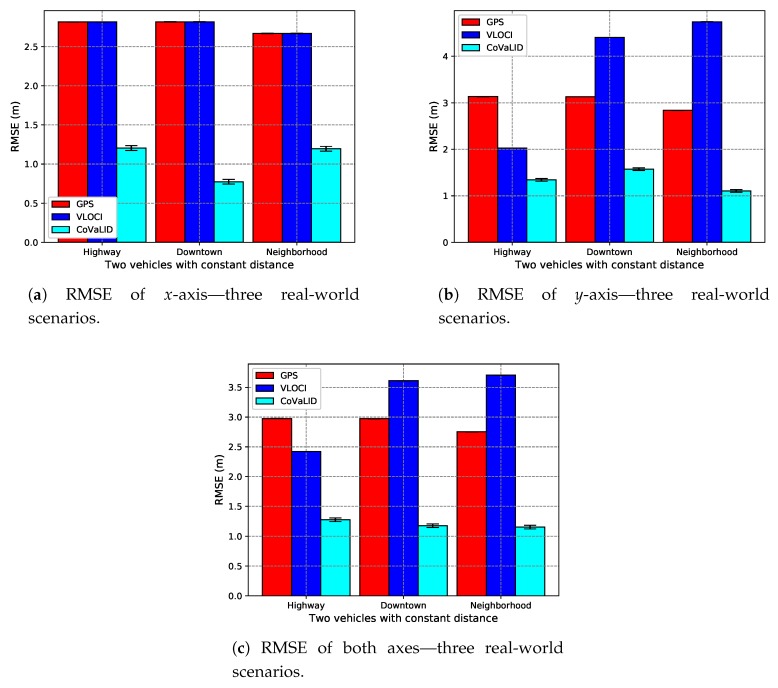
RMSE values in downtown, highway, and neighborhood scenarios—regarding the vehicle in a straight-line trajectory.

**Figure 14:**
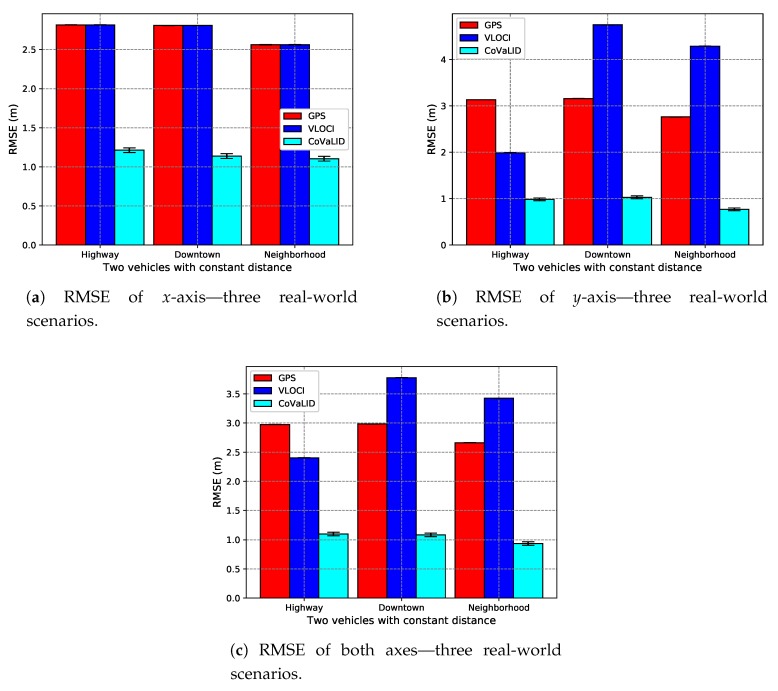
RMSE values in downtown, highway, and neighborhood scenarios—regarding the vehicle in a turn trajectory.

When compared straight-line against turning trajectory in the downtown scenario, in the *x*-axis, as depicted in Figure 13a and Figure 14a, we can notice that CoVaLID had better accuracy in the straight-line trajectory. The same occurred in the highway scenario, but with a just slightly better result when compared to the turning trajectory. On the other hand, in the neighborhood scenario, the turning trajectory had almost the same performance as in a straight-line scenario.

In the *y*-axis, we can notice that the behavior of CoVaLID in a straight-line trajectory was the opposite presented in the *x*-axis. As shown in Figure 13b, the RMSE values show that in the downtown scenario, the CoVaLID performance decreased, whereas, in both highway and neighborhood scenarios, the accuracy was improved. Regarding the VLOCI algorithm, only in highway scenarios, it can overcome the GPS accuracy. Surprisingly, in *y*-axis simulations and using trajectory with turns, the accuracy of CoVaLID was improved, as shown in Figure 14b. It can be explained because usually, the vehicle position given by GPS does not lie in the same line as the distance information provided by sensors which implies in an automatic triangle rotation when triangle similarity concepts are performed.

Overall, we can notice that all tested approaches presented similar behaviors for both trajectories simulated. As we can see in Figure 13c and Figure 14c, on average in both axes, CoVaLID had the best performance when compared to VLOCI, and GPS. On the other hand, VLOCI was able to overcome GPS only in highways scenarios. However, it is worth mentioning that the CoVaLID approach is dependable on the high quality of sensors information about distance among vehicles.

#### 4.4.3. The Impact of Distance Information Error

This section aims at analyzing and assessing the sensors that are suitable to provide the distance information in all tested scenarios. We used the sensor’s specifications provided in the literature [35]. The used parameters and their respective sensors are described in Table 7.

Moreover, all simulations in this section were conducted using 10 vehicles with both distance and velocity set randomly, 2 m of GPS error, and the scenarios were divided into random, straight-line, and trajectories with turns.

In a random trajectory scenario, the results presented in the *x*-axis show that CoVaLID had similar behavior for all three tested scenarios. We can observe that the higher is the distance information error, the worse is the CoVaLID performance. The same behavior can be seen in the *y*-axis, and as a consequence, on average of both axes. However, the CoVaLID accuracy just decreased its performance around 32 cm in the downtown scenario.

Also, *y*-axis overall, we noticed that in both downtown and neighborhood scenarios, the VLOCI behavior was affected similarly as CoVaLID, whereas in highway scenario the VLOCI kept its accuracy almost constant due to the distance measurement model used in VLOCI algorithm along with vehicles’ skewed position treatment. Another interesting point is that in downtown scenario was also the worst CoVaLID performance as expected since the buildings and other obstacles can affect the sensors’ measurements.

**Figure 15:**
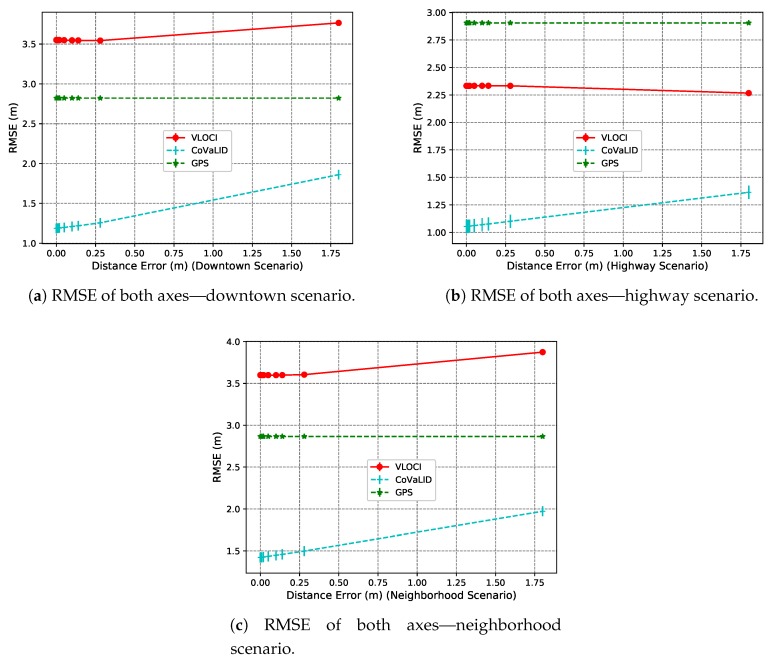
RMSE values of the average of both axes in downtown, highway, and neighborhood scenarios—regarding the distance error information.

The RMSE values on average of both axes, seen in Figure 15a–c can summarize the behavior of the tested approaches. Overall, we can notice that the best accuracy was reached in the highway scenario that is due to its characteristics: a scenario with no buildings or obstacles, and mostly a straight-line scenario. Moreover, results suggest that the EKF works well using the velocities of the vehicles in this scenario.

**Figure 16:**
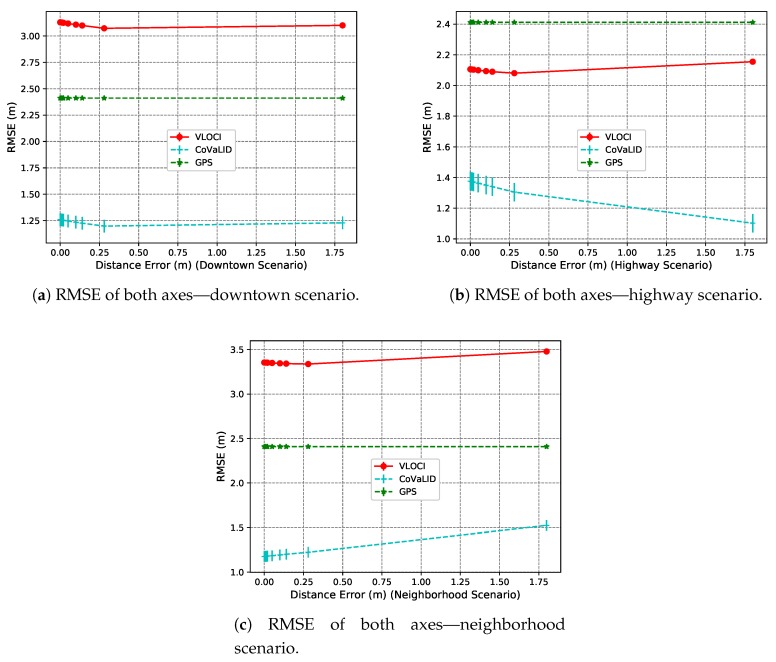
RMSE values of the average of both axes in downtown, highway, and neighborhood scenarios—regarding the distance error Information in a straight-line trajectory.

Using the straight-line trajectory, we can notice that, according to Figure 16a–c, in both downtown and highway scenarios, the CoVaLID improved its performance due to two reasons. First, because of the trajectory characteristics (a straight-line). Second, because the EKF deals well with noises in distance information in these scenarios along with higher velocities, as seen in the highway case. However, as expected, in the neighborhood scenario, the CoVaLID had the worst performance due to the lower vehicles’ velocity.

**Figure 17:**
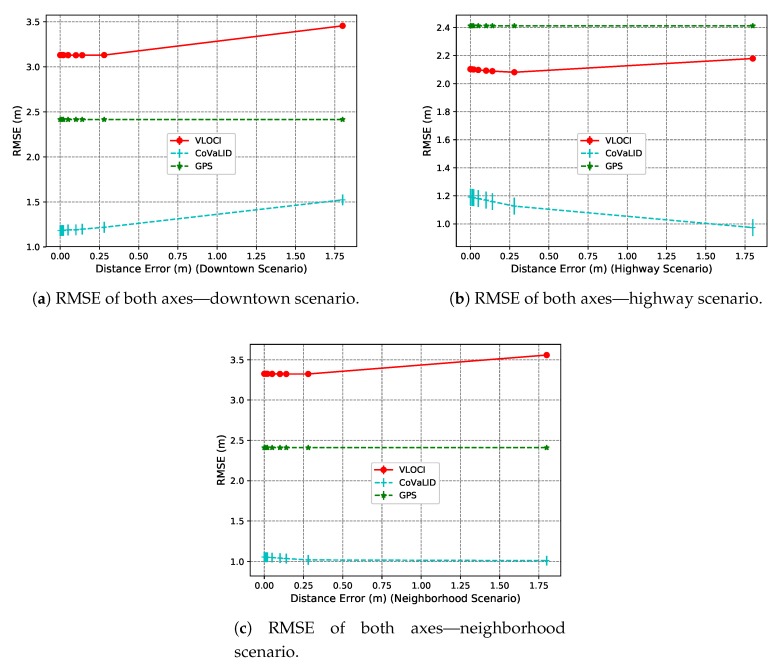
RMSE values of the average of both axes in downtown, highway, and neighborhood scenarios—regarding the distance error information in a trajectory with curves.

From trajectories with turns, we can observe, according to Figure 17a–c, that CoVaLID presented the same behavior as in the highway scenario, improving its performance. Whereas, in the downtown scenario, its accuracy was affected by the scenarios’ characteristics such as obstacles, lower vehicles’ velocity, and sensors’ field of view. On the other hand, in the neighborhood scenario, the CoVaLID kept RMSE values almost constant. It can be explained due to the combination of lower vehicles’ velocities and scenario characteristics.

Hence, we detailed the impact of the sensors used to provide distance information, and the results presented in this section suggest that either the trajectories and the noisy distance information can affect our proposed solution in some way.

#### 4.4.4. Sensors Analysis

In this work, we tested and analyzed three different sensors that are capable of providing distance information that is used in our proposed approach to improve the vehicles’ position estimation. As seen in Table 7, we used radars, lasers, and cameras as sensors.

Radar sensors are capable of measuring both the relative distance and speed of a target in short, medium, and long-range, with ranges up to 20 m, 100 m, and 250 m, respectively. In VANets, the radars commonly used to address localization problems are long-range sensors. In this section, we tested four different radars, all of them of long-range. Since most radar sensors have no moving parts, the across-track accuracy is affected. It can be verified through results shown in the previous section, wherein all tested scenarios, the accuracy was more affected in the *y*-axis than in the *x*-axis. However, these sensors work well even in challenging environmental conditions, such as rain, dust, and fog.

On the other hand, Laser sensors can measure the distance of an object. However, they are not able to measure the relative speed of a target with a single scan. For that purpose, lasers need successive scans. These kinds of sensors can be slightly more accurate than radars, although its accuracy is significantly affected by environmental conditions, and their prices are still higher when compared to radars. In this work, we tested two different laser sensors, and results show that the impact of the use of lasers is not significant in terms of accuracy. However, all applied approaches were not tested in adverse environmental conditions, and it is known [35] that it can affect the accuracy of distance information given by lasers.

The cameras can also be used as devices that provide reliable distance information since its accuracy is around 0.01 m, and also they have higher update rates when compared to the other sensors. Although, it is a range-limited sensor that operates in a range of up to 10 m, as described in Table 7. Furthermore, its accuracy can be affected when it is exposure to lights, mainly sunlight, and even at night when the other vehicles’ lights can interfere in the camera’s performance. In our simulation results, we can notice that cameras can have good accuracy since the distances among the target and the neighbors are up to 10 m. Also, the drawback situation with lights was not tested.

Overall, of all three sensors, the higher cost is the Laser, whereas the cheapest are the cameras, and both require a massive amount of data since they use 3D environmental representation. Hence, they are considered a high computational cost solution. On the other hand, radars cannot reach the resolution for object identification, but they can detect it.

Finally, analyzing the advantages and disadvantages of each sensor cited above, it is clear that it would be possible to combine the outputs of all three sensors in a data fusion approach to achieve a high level of accuracy since their efficiency depends on their field of view.

## 5. Conclusions

In this work, we have proposed an improvement to the BOuND algorithm, named CoVaLID, which improves GPS position of nearby vehicles and minimize their errors through an extended Kalman filter that performs the data fusion of both GPS and distance information to provide a precise estimation for the vehicle’s positions within the network area. Also, our solution takes advantage of a weighted average method to put more confidence in distance information given by neighbors closer to the target. We evaluated and tested our solution through simulations in three real-world scenarios, such as highway, downtown, and neighborhood.

Our results show that our solution can minimize the average error between the perfect position and the position given by GPS by 63%. In addition, our solution can estimate the node position better than when compared to the state-of-the-art VLOCI algorithm, in all real-world-tested scenarios using a fewer quantity of nodes as verified by RMSE and MAE values, in Section 4.4.1. Thus, the increasing of the number of vehicles did not severely affect our proposed solution due to the weighted average method applied in CoVaLID. Moreover, it is noticed that in a straight-line trajectory CoVaLID presented a better performance in the highway scenario, in the *x*-axis, whereas, in the *y*-axis, our proposed solution had similar RMSE values for all three tested scenarios. It is important to note that our solution focuses on GPS inaccuracies and not on GPS outages. Furthermore, the results in Section 4.4.3 support that CoVaLID can be affected by the distance information error, which is provided by sensors, such as radars, lidars, and cameras. Lastly, we presented an exploratory analysis of these three sensors, which describes the advantages and drawbacks of each sensor and how they could be used in different scenarios.

As future work, we will test our solution using different Bayesian statistical models. Furthermore, we will evaluate our proposed solution combining CoVaLID with vehicle-to-infrastructure (V2I) communication, where vehicles can communicate with a roadside unit (RSU). We also will extend our solution to solve the GPS outage problem in scenarios where GPS signal is not available, such as in tunnels.

## Figures and Tables

**Table 1:** Weights for the weighted average method.

Weights	Range
90%	for nodes up to 10 m of distance
80%	for nodes from 10.01 m to 20 m of distance
10%	for nodes from 20.01 m to 30 m of distance
1%	for nodes from 30.01 m of distance

**Table 2:** Simulation parameters.

Parameters	Value
Network Interface	Nic80211p
Communication Range	200 m
GPS error	1, 2, 5, 10 m
Number of Iterations	10
Number of Vehicles	from 2 to 10

**Table 3:** Localization techniques accuracy.

Localization Techniques	RMSE	MAE	RMSE	MAE
	*x*-Axis	*x*-Axis	*y*-Axis	*y*-Axis
CoVaLID without RC	1.871733 m	1.54864 m	2.633068 m	2.18359 m
CoVaLID + RC	1.006493 m	0.997129 m	2.067209 m	1.64502 m
VLOCI	2.710796 m	2.388535 m	2.238849 m	1.850759 m
GPS	2.710796 m	2.388535 m	2.672965 m	2.2277 m

**Table 4:** Localization techniques accuracy for 1 m of GPS error.

*Localization Techniques*	*RMSE*	*MAE*	*RMSE*	*MAE*
	*x-Axis*	*x-Axis*	*y-Axis*	*y-Axis*
CoVaLID	1.007360 m	0.908047 m	0.829160 m	0.660428 m
VLOCI	1.084340 m	0.955464 m	0.882375 m	0.725475 m
GPS	1.084340 m	0.955464 m	1.069160 m	0.891048 m

**Table 5:** Localization techniques accuracy for 5 m of GPS error.

*Localization Techniques*	*RMSE*	*MAE*	*RMSE*	*MAE*
	*x-Axis*	*x-Axis*	*y-Axis*	*y-Axis*
CoVaLID	1.000024 m	0.989990 m	4.158237 m	3.266210 m
VLOCI	5.421584 m	4.777160 m	4.588680 m	3.844891 m
GPS	5.421584 m	4.777160 m	5.345863 m	4.455358 m

**Table 6:** Localization techniques accuracy for 10 m of GPS error.

*Localization Techniques*	*RMSE*	*MAE*	*RMSE*	*MAE*
	*x-Axis*	*x-Axis*	*y-Axis*	*y-Axis*
CoVaLID	1.256964 m	1.150331 m	7.532818 m	6.169233 m
VLOCI	10.343913 m	8.674089 m	7.802703 m	6.169344 m
GPS	10.343913 m	8.674089 m	10.596546 m	9.417037 m

**Table 7:** Sensors specification.

Sensor Type	Sensor Brand	Range	Distance Measuring Accuracy
Camera	SwissRanger SR4000	10 m	±0.01 m
Laser	Velodyne HDL-64E S2	120 m	±0.02 m
Laser	Quanergy M8-1	150 m	±0.05 m
Radar	Bosh LRR3	250 m	±0.10 m
Radar	Continental ARS30x	250 m	±0.14 m
Radar	SMS UMRR Type40	250 m	±0.28 m
Radar	Delphi ESR	174 m	±1.80 m

**Figure A1:**
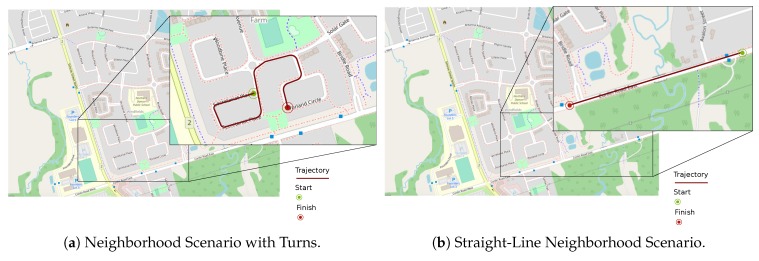
Trajectories in the neighborhood scenario.

**Figure A2:**
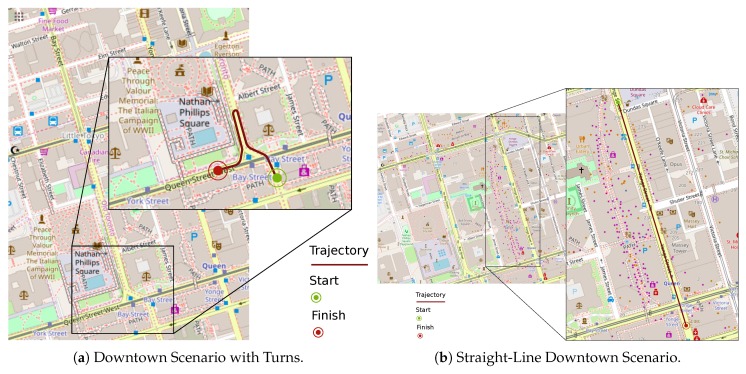
Trajectories in the downtown scenario.

**Figure A3:**
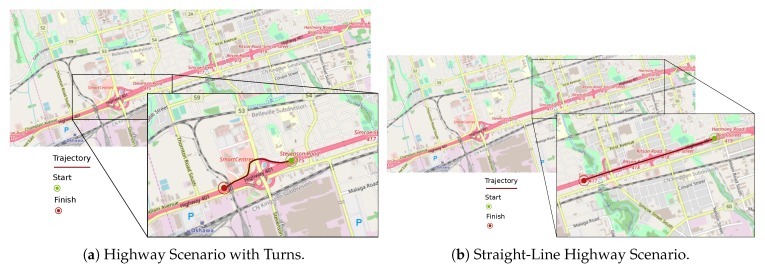
Trajectories in the highway scenario.

## Appendix A

This appendix describes the trajectories used in Section 4.4.2.
